# C4OH is a potential newborn screening marker—a multicenter retrospective study of patients with beta-ketothiolase deficiency in China

**DOI:** 10.1186/s13023-021-01859-5

**Published:** 2021-05-17

**Authors:** Yiming Lin, Zhantao Yang, Chiju Yang, Haili Hu, Haiyan He, Tingting Niu, Mingfang Liu, Dongjuan Wang, Yun Sun, Yuyan Shen, Xiaole Li, Huiming Yan, Yuanyuan Kong, Xinwen Huang

**Affiliations:** 1 Department of Genetics and Metabolism, Children’s Hospital, Zhejiang University School of Medicine, National Clinical Research Center for Child Health, 3333 Binsheng Road, Hangzhou, 310052 China; 2 Neonatal Disease Screening Center, Quanzhou Maternity and Children’s Hospital, Quanzhou, China; 3 Continuing Medical Education and Research Center, Dian Diagnostics Group Co., Ltd, 329 Jinpeng Street, Xihu District, Hangzhou, 310030 China; 4 Jining Maternal and Child Health Family Service Center, Jining, China; 5 Neonatal Disease Screening Center, Hefei Maternal and Child Health, Family Planning Service Center, Anhui, China; 6 Wuhu Maternal and Child Health Family Planning Service Center, Anhui, China; 7 Shandong Provincial Maternal and Child Health Care Hospital, Shandong, China; 8 Liaocheng Maternal and Child Health Hospital, Shandong, China; 9 Center for Clinical Molecular Medicine/Newborn Screening Center, Children’s Hospital, Chongqing Medical University, Chongqing, China; 10 Nanjing Maternity and Child Health Care Hospital, Women’s Hospital of Nanjing Medical University, Jiangsu, China; 11 Neonatal Disease Screening Center, Huaihua Maternal and Child Health Hospital, Huaihua, China; 12 Third Affiliated Hospital of Zhengzhou University, Henan, China; 13 Department of Genetic Medicine, Hunan Provincial Maternal and Child Health Care Hospital, Changsha, Hunan China; 14 Department of Newborn Screening, Beijing Obstetrics and Gynecology Hospital, Capital Medical University, 251 Yaojiayuan Road, Chaoyang District, Beijing, 100026 China

**Keywords:** Beta-ketothiolase deficiency, Chinese, Newborn screening, *ACAT1*, Isoleucine catabolism

## Abstract

**Background:**

Beta-ketothiolase deficiency (BKTD) is an autosomal recessive disorder caused by biallelic mutation of *ACAT1* that affects both isoleucine catabolism and ketolysis. There is little information available regarding the incidence, newborn screening (NBS), and mutational spectrum of BKTD in China.

**Results:**

We collected NBS, biochemical, clinical, and *ACAT1* mutation data from 18 provinces or municipalities in China between January 2009 and May 2020, and systematically assessed all available published data from Chinese BKTD patients. A total of 16,088,190 newborns were screened and 14 patients were identified through NBS, with an estimated incidence of 1 per 1 million newborns in China. In total, twenty-nine patients were genetically diagnosed with BKTD, 12 of which were newly identified. Most patients exhibited typical blood acylcarnitine and urinary organic acid profiles. Interestingly, almost all patients (15/16, 94%) showed elevated 3-hydroxybutyrylcarnitine (C4OH) levels. Eighteen patients presented with acute metabolic decompensations and displayed variable clinical symptoms. The acute episodes of nine patients were triggered by infections, diarrhea, or an inflammatory response to vaccination. Approximately two-thirds of patients had favorable outcomes, one showed a developmental delay and three died. Twenty-seven distinct variants were identified in *ACAT1*, among which five were found to be novel.

**Conclusion:**

This study presented the largest series of BKTD cohorts in China. Our results indicated that C4OH is a useful marker for the detection of BKTD. The performance of BKTD NBS could be improved by the addition of C4OH to the current panel of 3-hydroxyisovalerylcarnitine and tiglylcarnitine markers in NBS. The mutational spectrum and molecular profiles of *ACAT1* in the Chinese population were expanded with five newly identified variants*.*

**Supplementary Information:**

The online version contains supplementary material available at 10.1186/s13023-021-01859-5.

## Background

Beta-ketothiolase deficiency (BKTD, OMIM #203750) is an autosomal recessive disorder caused by a defect in mitochondrial acetoacetyl-CoA thiolase (T2, EC 2.3.1.9) that affects both isoleucine catabolism and ketolysis [1–3]. This disease is clinically characterized by intermittent episodes of ketoacidosis. The T2 encoding gene *ACAT1* is located on chromosome 11q22.3–23.1 and consists of 12 exons spanning approximately 27 kb. Characteristic laboratory findings include marked ketonuria and elevated urinary excretion of isoleucine catabolic intermediates, such as 2-methyl-3-hydroxybutyrate (2M3HB), tiglylglycine (TIG), and 2-methylacetoacetate (2MAA). Notably, 2MAA is unstable and is therefore difficult to detect by gas chromatography-mass spectrometry, especially in non-fresh urine samples.

BKTD is included in newborn screening (NBS) programs in many countries, and 3-hydroxyisovalerylcarnitine (C5OH) and tiglylcarnitine (C5:1) are the primary screening markers [4]. Patients with BKTD commonly have elevated levels of C5OH and C5:1. However, normal acylcarnitine profiles have been reported in some patients, even during acute metabolic crises [5, 6]. Therefore, NBS for BKTD can be challenging as some patients fail to be identified, indicating that the use of only two markers, C5OH and C5:1, is insufficient for BKTD NBS. However, it remains challenging to detect BKTD even using post-analytic interpretative tools, further indicating the complexity of early diagnosis of BKTD [6, 7].

Since the first description of BKTD in 1971, approximately 250 patients have been reported worldwide [1]. While several retrospective studies have investigated BTKD patients of various ethnic backgrounds [8–11], there is little information available regarding the incidence, NBS, and mutational spectrum in China. We encountered a case of genetically diagnosed BKTD with increased 3-hydroxybutyrylcarnitine (C4OH) levels (the cutoff is set at the 99.5 centile) but normal C5OH and C5:1 profiles at the time screening, and no increased 2M3HB was detected in the urine, even during acute decompensation. To further evaluate the significance of C4OH in BKTD, we launched a multicenter national cohort study through the Zhejiang Neonatal Disease Screening Center, a unit of the China Neonatal Screening Group. The specific objectives of the study were: (a) to investigate the baseline levels of amino acids and acylcarnitines in BKTD; (b) to evaluate the importance of C4OH, along with C5OH and C5:1 in BKTD screening; and (c) to further understand the incidence, clinical features, genetic features, and prognosis of BKTD. We systematically reviewed the available BKTD clinical reports that included the Chinese population [12–16] and retrospectively analyzed the biochemical, clinical, and molecular features of 29 Chinese BKTD patients from our NBS and selective metabolic screening (SMS) data. Previously, C4OH was primarily used to evaluate the metabolic profile of BKTD; however, elevated levels of C4OH can often be observed in our patients during NBS. Thus, we propose that C4OH is a potential marker for BKTD screening. Additionally, we identified five novel variants among a total of 27 distinct variants of *ACAT1* in the Chinese population.

## Results

### BKTD NBS and acylcarnitine analysis

In this cohort, 29 Chinese patients were genetically diagnosed with BKTD, 14 of which were diagnosed through NBS while 17 were previously reported [12–16]. During the study period, a total of 16,088,190 newborns were screened. BKTD was detected in six of the 18 provinces or municipalities involved. The overall incidence of BKTD was 1 in 1,149,156 births (Table 1). The median C4OH concentration was 1.38 ± 0.94 μmol/L (range 0.26–3.58 μmol/L, reference value 0.02–0.3 μmol/L). Among the 16 patients with available C4OH levels, 15 (94%) showed an increase. The median C5OH concentration was 1.36 ± 0.87 μmol/L (range 0.44–3.4. μmol/L, reference value 0.06–0.5. μmol/L). The median C5:1 concentration was 0.37 ± 0.28 μmol/L (range 0.02–1.22 μmol/L, reference value: 0–0.05 μmol/L). Almost all patients (23/25, 95%) exhibited elevated C5OH and C5:1 levels. Notably, patients No. 4 and 11 had increased C4OH levels only, while C5OH and C5:1 levels were normal, even during the course of metabolic decompensations. However, as described in the case report, C4OH levels in patient No. 4 returned to normal when recalled two weeks after the initial NBS, indicating that C4OH may be normal in the healthy state. In contrast, patient No. 10 had increased C5OH and C5:1 levels, but normal C4OH levels. In the available data, all other patients showed simultaneous elevation of C4OH, C5OH, and C5:1 at the time of NBS (Additional file 1: Table S1 and Additional file 2: Table S2). The data in the SMS group is relatively incomplete since data quality of clinical patients was heterogeneous and most of the data were retrieved from previous reports. Comparing the statistic values of C4OH, C5OH, and C5:1 between the NBS and SMS groups, there was no statistic difference (*P* > 0.05 each).

**Table 1:** Data of newborn screening for BKTD

Province/municipality	Screened newborns	Confirmed BKTD	Incidences
Zhejiang	3,830,012	4	1:957,503
Shandong	3,060,547	4	1:765,137
Jiangsu	2,240,078	2	1:1,120,039
Hunan	1,400,320	1	1:1,400,320
Shanghai	1,230,125	2	1:615,063
Fujian	977,173	0	0
Henan	879,231	0	0
Guangdong	598,007	0	0
Anhui	560,000	1	1:560,000
Gansu	500,789	0	0
Jiangxi	250,010	0	0
Jilin	156,126	0	0
Shanxi	150,003	0	0
Chongqing	78,096	0	0
Beijing	70,235	0	0
Sichuan	60,170	0	0
Hainan	30,012	0	0
Yunnan	17,256	0	0
Total	16,088,190	14	1:1,149,156

### Biochemical and clinical features

Urinary organic acid results were available for 27 patients. Almost all patients exhibited a characteristic increase in urinary 2M3HB and TIG, except for patient No. 4, who only had increased urinary 3-hydroxybutyric acid, but no increase in 2M3HB and TIG during acute decompensation. This urinary organic acid pattern was consistent with the observation of only C4OH levels being increased when examined by MS/MS.

This cohort of 29 patients included four pairs of siblings, 15 males and 9 females, and the gender of the remaining five patients was not described. Eighteen patients (18/29, 62%) presented with clinical symptoms, including hypotonia, fever, vomiting, tachypnea, seizures, neurological impairment, and metabolic acidosis. Of these, two patients presented with acute metabolic decompensations during the neonatal period, 13 displayed clinical symptoms beyond the neonatal period (mean 10.5 months), and the remaining three had no reported onset time. The acute episodes of nine patients were triggered by infections, diarrhea, or the inflammatory response to vaccination. Nineteen of 23 individuals (82% of the cohort) for whom there was information had a favorable outcome, while one showed a developmental delay and three died. For the remaining six patients, there was no information on their outcome, but all had clinical manifestations. Detailed information on the biochemical and clinical manifestations of the 29 BKTD patients is summarized in Additional file 1: Table S1.

### Molecular findings

All 29 patients harbored compound heterozygous or homozygous *ACAT1* variants. Twenty-seven distinct variants were identified, among which 51.9% (14/27) were missense variants, 22.2% (6/27) were frameshift variants, 14.8% (4/27) affected splicing, 7.4% (2/27) were nonsense variants, and 3.7% (1/27) were a large deletion. Twenty-two of these *ACAT1* variants have been previously described, and the other five were novel (Table 2). The novel variants are c.1119dup (p.V374Sfs*86), c.631C > A (p.Q211K), c.1154A > T (p.H385L), c.401 T > C (p.M134T), and c.481 T > C (p.Y161H). The most common variant in this cohort was c.622C > T (p.R208*) with a frequency of 17.2%, followed by c.1006-1G > C (8.6%) and c.1124A > G (p.N375S) (8.6%). In addition, c.419 T > G (p.L140R) and c.997G > C (p.A333P) were relatively common (Table 2).

**Table 2:** The detected *ACAT1* variants and their frequencies in Chinese patients

Nos	Variants	Locations	Mutant allele (no.)	Frequencies (%)	ClinVar (clinical significance)	HGMD	References
1	c.622C > T (p.R208*)	Exon 7	10	17.2	P	CM102337	Fukao et al. [27] and Nguyen et al. [11]
2	c.1006-1G > C	Intron 10	5	8.6	P	CS920725	Fukao et al. [27] and Nguyen et al. [11]
3	c.1124A > G (p.N375S)	Exon 11	5	8.6	P	CS083860	Fukao et al. [29]
4	c.419 T > G (p.L140R)	Exon 5	4	6.9	NF	NF	Xu et al. [15]
5	c.997G > C (p.A333P)	Exon 10	4	6.9	P/LP	CM950009	Su et al. [13]
6	c.121-3C > G	Intron 2	3	5.2	VUS	NF	Su et al. [13]
7	c.653C > T (p.S218F)	Exon 7	3	5.2	LP	NF	Wen et al. [14]
8	c.72 + 1G > A	Intron 1	2	3.4	NF	NF	Xu et al. [15]
9	c.373G > T (p.V125F)	Exon 5	2	3.4	NF	NF	Xu et al. [15]
10	exon 6-12del	Exon 6	2	3.4	NF	NF	Xu et al. [15]
11	c.631C > A (p.Q211K)	Exon 7	2	3.4	NF	NF	This study
12	c.83_84del (p.Y28Cfs*38)	Exon 2	1	1.7	P	CD971964	Paquay et al. [8] and Su et al. [13]
13	c.163 T > A (p.F55I)	Exon 3	1	1.7	NF	CX102338	NR
14	c.229del (p.E77Kfs*10)	Exon 3	1	1.7	NF	NF	Xu et al. [15]
15	c.238 + 1G > A	Intron 3	1	1.7	NF	NF	Yang et al. [12]
16	c.354_355delinsG (p.C119Vfs*4)	Exon 5	1	1.7	P	NF	Law et al. [16]
17	c.401 T > C (p.M134T)	Exon 5	1	1.7	NF	NF	This study
18	c.481 T > C (p.Y161H)	Exon 6	1	1.7	NF	NF	This study
19	c.642 T > G (p.Y214*)	Exon 7	1	1.7	P	NF	NR
20	c.721dup (p.T241Nfs*14)	Exon 7	1	1.7	NF	NF	Yang et al. [12]
21	c.756_758del (p.E252del)	Exon 8	1	1.7	LP	CD076722	Sakurai et al. [3]
22	c.829A > C (p.T277P)	Exon 9	1	1.7	VUS	NF	Su et al. [13]
23	c.890C > A (p.T297K)	Exon 9	1	1.7	VUS/LP	CM950007	Su et al. [13]
24	c.928G > C (p.A310P)	Exon 9	1	1.7	NF	NF	Yang et al. [12]
25	c.1119dup (p.V374Sfs*86)	Exon 11	1	1.7	NF	NF	This study
26	c.1154A > T (p.H385L)	Exon 11	1	1.7	NF	NF	This study
27	c.1163G > T (p.G388V)	Exon 11	1	1.7	P	NF	Paquay et al. [18]

NF, not found; NR, not reported; VUS, variants of uncertain clinical significance; P, pathogenic; LP, likely pathogenic

^a^The previously unreported novel variants of this study are in boldface type

^b^HGMD: http://www.hgmd.cf.ac.uk/ac/index.php

^c^ClinVar: https://www.ncbi.nlm.nih.gov/clinvar/

**Fig. 1:**
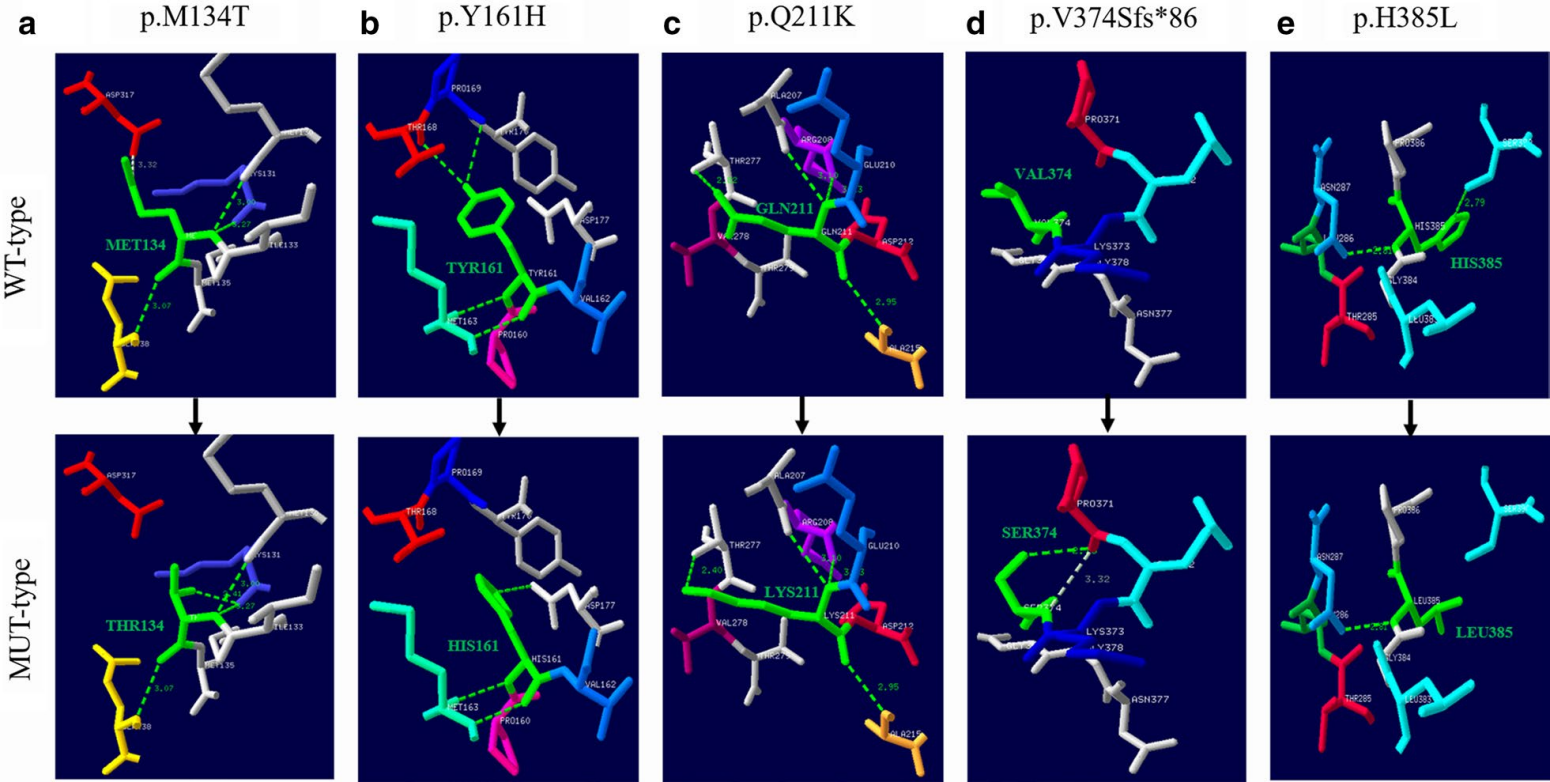
Three-dimensional structure analysis modeling of wild-type and mutant *ACAT1* protein products. Green dashed lines represent hydrogen bonds and the green number shows the hydrogen bond distances; WT-type: Wild-type, MUT-type: Mutation-type. **a** The p.M134T variant may alter the side chain conformations of residues by inducing intramolecular hydrogen bonding with 131-LYS and losing intramolecular hydrogen bonding with 317-ASP. **b** The p.Y161H variant may alter the side chain conformations of residues by inducing intramolecular hydrogen bonding with 177-ASP and losing intramolecular hydrogen bonding with 168-THR and 169-PRO. **c** The p.Q211K variant may alter the side chain conformations of residues by increasing the intramolecular hydrogen bonding distance with 277-THR. **d** The p.V374Sfs*86 variant may alter the side chain conformations of residues by inducing intramolecular hydrogen bonding with 371-PRO and produces truncated proteins lacking the conserved domains. **e** The p.H385L variant may alter the side chain conformations of residues through the loss of intramolecular hydrogen bonding with 390-SER

None of these novel variants were recorded in disease databases such as ClinVar and HGMD, and they were not detected in the control group. The novel variants were not present or had extremely low allelic frequencies in the dbSNP, ExAC, 1000 Genome, and GnomeAD databases. In silico analysis suggested that all novel variants were potentially pathogenic (Additional file 3: Table S3). Structural modeling revealed that the c.401 T > C (p.M134T) variant may alter the side chain conformations of the affected residues by inducing a new intramolecular hydrogen bond with 131-LYS and losing intramolecular hydrogen bonding with 317-ASP. The c.481 T > C (p.Y161H) variant may alter the side chain conformations of the affected residues by inducing intramolecular hydrogen bonding with 177-ASP and losing intramolecular hydrogen bonding with 168-THR and 169-PRO. The c.631C > A (p.Q211K) variant may alter the side chain conformations of the affected residues by increasing the intramolecular hydrogen bonding distance with 277-THR. The c.1119dup (p.V374Sfs*86) variant may alter the side chain conformations of the affected residues by inducing intramolecular hydrogen bonding with 371-PRO, leading to a truncated protein lacking the conserved domains. The c.1154A > T (p.H385L) variant may alter the side chain conformations of the affected residues through the loss of intramolecular hydrogen bonding with 390-SE (Fig. 1).

## Discussion

To the best of our knowledge, this is the largest BKTD cohort study in China. The large number of screened newborns in this study provided a more comprehensive perspective on the incidence of BKTD detected via NBS in China. Biochemical, clinical, and molecular features of Chinese patients with BKTD were summarized, contributing to NBS, early diagnosis, and timely treatment of this rare disease.

Few studies regarding the incidence of BKTD have been reported. The incidence is estimated to be 1:190,000 in northern Vietnam [11], 1:313,000 in North Carolina, and 1:232,000 in Minnesota in the United States [5, 17]. Our study investigated, for the first time, over 16 million newborns and identified 14 BKTD patients with a minimal estimate of the incidence of approximately 1:1,000,000 in China. The incidence is lower than those reported in other studies [5, 11, 17], and this difference may be due to differences in ethnic backgrounds and awareness of the disease. It should be noted that the incidence reported here may not be sufficiently accurate given that the other 15 patients were identified by SMS, and the data for these patients were incomplete and therefore difficult to interpret. We should also note that the false negatives (FN) seem to be inevitable for BKTD NBS, and the missed cases would not have come to our attention. Furthermore, the actual incidence of BKTD may be higher in China because some early neonatal deaths and patients who suffered mild episodes may not have been identified.

From a clinical perspective, BKTD is an ideal disease for NBS. However, FN results have been reported in several NBS programs [5, 6, 18]. Although both C5OH and C5:1 acylcarnitines are well-known markers used for BKTD screening, the levels of these two markers are not necessarily high during NBS, or even during acute metabolic crisis. In our study, two patients (Nos. 4 and 11) had only increased C4OH but not C5OH and C5:1, even in the period of acute decompensation. However, we also found that one patient (No. 10) exhibited increased levels of C5OH and C5:1, but no increase in C4OH. Notably, the C4OH level in patient No. 4 was significantly elevated during NBS but returned to normal when recalled, and then increased again at 11 months, indicating that C4OH can be variable and may be normal when the patient is in a stable condition. Beta-ketothiolase not only functions during ketone body utilization (ketolysis) by catalyzing the thiolytic cleavage of acetoacetyl-coenzyme A to produce two molecules of acetyl CoA in extrahepatic tissues, but also catalyzes the conversion of 2-methylacetoacetyl-coenzyme A during isoleucine catabolism. D-3-hydroxybutyrate ketone bodies can be converted into D-3-hydroxybutyrylcarnitine (C4OH) in vivo and in vitro [19]. Thus, it is reasonable that there is not only an increase in C5OH and C5:1 but also an increase in C4OH in BKTD patients. Taken together, we speculate that some BKTD patients may be overlooked if any of the three acylcarnitines (C5OH, C5:1, and C4OH) is used as an independent screening marker. Conversely, FN results can be reduced by using several markers and/or combinations of them. Therefore, our study therefore strongly suggests that C4OH is a very useful and powerful marker for the detection of BKTD that can be used as a co-primary marker along with elevated levels of C5OH and C5:1 for BKTD screening. The performance of BKTD NBS may be improved by adding C4OH to the C5OH and C5:1 combination in NBS.

In this study, although almost all patients (except patient No. 4) exhibited a characteristic increase in urinary 2M3HB and TIG, both urinary markers may be undetectable in the healthy state and only detectable during decompensations. Sometimes, urinary organic acid profiles are not typical, as appeared in inpatient No. 4. Enzyme assays or DNA-based genetic testing is therefore necessary to confirm BKTD. In addition, elevated excretion of urinary 2M3HB and TIG indicates not only BKTD but also HSD10 mitochondrial disease (HSD10MD, OMIM #300438), which is a rare X-linked recessive disorder caused by a hemizygous or heterozygous mutation in the *HSD17B10* gene [20]. Fukao et al. reported a 6-year-old Japanese boy who was initially diagnosed with BKTD based on metabolic profiling; however, enzyme activity assays and mutation analysis later confirmed that the patient had HSD10MD [21]. Grunert and Sass recently described two patients who may actually have HSD10MD but were misdiagnosed with BKTD in earlier reports [1]. Thus, the diagnosis of BKTD cannot be based solely on metabolite data. Given the confusing blood acylcarnitine and urinary organic acid profiles between the two disorders, enzyme activity assays or mutation analysis are essential for differential diagnosis.

Most symptomatic patients in this cohort presented with acute metabolic decompensations or displayed neurologic impairment. Similar to the results of Grunert and Sass [1], neonatal presentation was rare in our study cohort appearing in only two patients (one previously reported and one newly reported). Abdelkreem et al. recently reported ten Indian patients presenting with episodes of ketoacidosis of variable severity, of whom six patients had a favorable outcome, while three developed neurodevelopmental sequelae and one died [9]. Similarly, approximately two-thirds of our cohort of patients had a favorable outcome, while three clinical patients identified by SMS presented with acute metabolic decompensations and died early. These data demonstrate that patients who did not undergo NBS had a delayed diagnosis and poorer prognosis. Early diagnosis of BKTD remains challenging, further emphasizing the importance of NBS for BKTD. NBS may be the only method for early detection of BKTD, and severe metabolic crises and death could be avoided if patients are properly managed. It is well known that acute episodes in most patients with BKTD are associated with infections. Consistent with previous studies, acute episodes in six of our patients were triggered by respiratory tract infections or diarrhea [4]. Notably, we observed that two patients developed severe metabolic crises apparently triggered by the inflammatory response to vaccination, with one resultant death. Therefore, vaccines should be administered more cautiously to BKTD patients given that metabolic abnormalities can be significantly worsened by the physiologic changes associated with the inflammatory response to vaccination. Poor feeding, vomiting, and diarrhea may be the clinically recognized symptoms of the inflammatory response or the signs of the decompensation [22, 23]. The severe reaction to a vaccine can appear clinically identical to the symptoms of a metabolic decompensation of a BKTD patient, leading to delays in recognition and management of the metabolic disorder.

At least 105 *ACAT1* variants associated with BKTD have been described so far [24]. Most are familial variants, and only four variants have been identified in more than six families. The most frequent variant, c.622C > T (p.R208*), was found in 28 families, most of which were of Vietnamese origin [24–26]. This variant was detected in six families and ten individuals in our patient cohort, and accounted for 17.2% of all mutant alleles identified in Chinese patients. This study indicated that c.622C > T (p.R208*) was the most common variant in Chinese patients, which is consistent with a previous study showing that it was a founder mutation in the Vietnamese population [11]. The second most common variant, c.1006-1G > C, a splice site variant associated with exon 11 skipping, was detected in 13 families, most of which were also Vietnamese [24, 27]. This variant was identified in four families in this cohort and, in line with previous studies [11, 24], was the second most common variant in Chinese patients. Two other common variants, c.578 T > G (p.M193R) and c.455G > C (p.G152A), were not observed in Chinese patients [9, 28]. Notably, another common variant in our cohort was c.1124A > G (p.N375S) and it was rarely identified in other populations. This variant activates a cryptic splice donor site and causes aberrant splicing [9, 24, 29]. Thus, the *ACAT1* mutational spectrum appears to vary among different ethnic groups.

This study identified five previously unreported variants, expanding the molecular profile of *ACAT1.* All these variants were predicted to be potentially pathogenic by in silico analyses. Structural modeling was also performed to augment the prediction results and revealed that all these *ACAT1* variants could disrupt the quaternary structure of T2 and potentially affect protein function. Further functional studies are necessary to confirm the pathogenicity of these variants.

Regarding the relationship between genotype and phenotype, previous studies have shown that the genotype and clinical phenotype do not correlate in BKTD. Patients with BKTD may have variable clinical phenotypes even if they have identical genotypes and similar environmental factors [1, 10, 11, 24]. Although genotype may affect the biochemical phenotype of BKTD patients, it is difficult to analyze the relationship between genotype and biochemical phenotype due to a lack of functional studies in a large number of familial *ACAT1* variants. Consistent with previous studies [1, 24], there was no obvious correlation between genotype and phenotype in this cohort of patients, including the biochemical features, age of onset, severity, and eventual outcome. There is insufficient information from this retrospective analysis to determine if some of the outcome variability is related to differences in management and timeliness of interventions after initial signs of decompensations.

## Conclusions

In summary, this study revealed, for the first time, that the incidence of BKTD in China is approximately 1 per 1 million newborns. Most patients have a favorable outcome, but severe metabolic decompensation and even death can occur. NBS is an effective method for identifying BKTD early and preventing severe metabolic crises. C4OH is a potential screening marker; the performance of BKTD NBS can be improved and FN results can be reduced by adding C4OH to C5OH and C5:1 for combination screening in NBS. The mutational spectrum of *ACAT1* in the Chinese population was established and five previously unreported novel variants were identified, expanding the molecular profile of *ACAT1.*

## Methods

### BKTD case report

A female newborn (patient no. 4 in Additional file 1: Table S1), now 23 months old, was found with increased C4OH (2.19 μmol/L, reference value 0.02–0.3 μmol/L) by MS/MS only at NBS. Her C4OH level returned to normal after two weeks when recalled, and thus she was released with the regular process of NBS. She presented with fever at 11 months of age, severe acidosis, and drowsiness after injection of the meningococcal vaccine and was admitted to the pediatric ICU. Her C4OH level at that time was 1.28 μmol/L and only increased 3-hydroxybutyric acid (ketone body) was identified through urine organic acid analysis. Genetic diagnosis revealed that this patient had compound heterozygous pathogenic variants c.163 T > A (p.55I) and c.1119dup (p.V374Sfs*86) in *ACAT1* which were confirmed to be inherited from both parents; thus, the patient was diagnosed with BKTD but required further enzymatic confirmation. At 1-year-old, she was hospitalized again due to fever, acidosis, somnolence, and metabolic disorder with increased C4OH level at 1.18 μmol/L. Although her condition stabilized after emergency treatment, she had irreversible mental and motor retardation. In this case, regardless of whether it was during the time of NBS or during acute metabolic crises, only increased C4OH, and not the characteristic pattern of BKTD, was observed. This special case prompted us to further investigate whether C4OH is a potential marker to be used, similarly to C5OH and C5:1, for BKTD screening.

### BKTD NBS

Participating centers from 18 provinces and municipality cities were selected nationally with strict criteria from the China Neonatal Screening Group. The selected centers cover seven national administrative regions, including Northwest, Northeast, East, Middle, South, Southwest, and North China. Each center had more than 15,000 accumulated newborns screened by MS/MS. NBS for BKTD was performed in each NBS center, and the procedure of NBS has been described in detail in our previous article [30]. The analytes, including C4OH, C5OH, and C5:1, were quantitated using MS/MS, and internal quality controls were used in each sample batch. The results were evaluated by the addition of two in-house quality control specimens per 96-well microplate for MS/MS analysis. External quality control programs from the China National Center for Clinical Laboratories (NCCL) and the Centers for Disease Control and Prevention (CDC) were also included for yearly evaluations. This study was approved by the Ethical Committee of Children’s Hospital, Zhejiang University School of Medicine (reference number: 2018-IRB-077) and was performed in accordance with the Declaration of Helsinki. Written informed consent was obtained from the parents of all patients.

### Study population

**Fig. 2:**
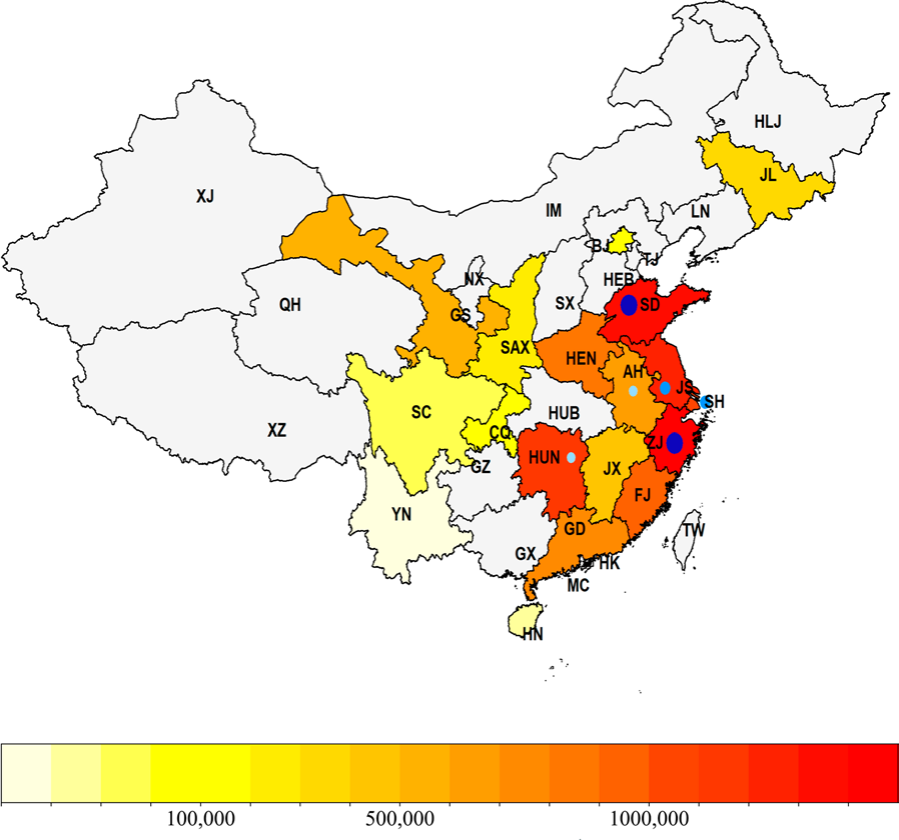
The distribution and sample size of the study population at each province/municipality in China. HLJ, Heilongjiang; JL, Jilin; LN, Liaoning; XJ, Xinjiang; IM, Inner Mongolia; BJ, Beijing; TJ, Tianjin, HEB, Hebei; SX, Shanxi; SAX, Shaanxi; NX, Ningxia; GS, Gansu; QH, Qinghai; SD, Shandong; JS, Jiangsu; AH, Anhui; HEN, Henan; SH, Shanghai; HUB, Hubei; CQ, Chongqing; SC, Sichuan; ZJ, Zhejiang; JX, Jiangxi; HUN, Hunan; GZ, Guizhou; YN, Yunnan; FJ, Fujian; TW, Taiwan; GD, Guangdong; GX, Guangxi; HN, Hainan, HK, Hongkong; MC, Macau. Gray areas indicate that no screening data were included. The darker the color, the larger the sample size. Blue circles represent the detection of positive BKTD cases with genetic confirmation

The study cohort covered NBS and reports between January 2009 and May 2020 (Fig. 2). Patients genetically diagnosed with BKTD (compound heterozygous or homozygous for *ACAT1* variants) were included. All cases of genetically confirmed Chinese BKTD patients published previously were reviewed and included in the study. Data on Chinese BKTD patients were retrieved from PubMed (https:www.ncbi.nlm.nih.gov/pubmed) by searching the keywords: beta-ketothiolase deficiency, β-ketothiolase deficiency, T2 deficiency, mitochondrial acetoacetyl-CoA thiolase deficiency, MAT deficiency, or 2-methylacetoacetyl-coenzyme A thiolase deficiency; Chinese or China; and *ACAT1*.

### Genetic diagnosis and data analysis

Disease incidence was calculated by dividing the number of BKTD patients diagnosed via NBS by the total number of screened newborns. The acylcarnitine profiles, clinical information, and biochemical and genetic testing results were collected for analysis. Data of the acylcarnitine profiles in the NBS and SMS groups were tested using unpaired two-tailed *t*-test, statistical evaluation were performed using SPSS 22.0 version (SPSS Inc., Chicago, IL, USA). Differences between groups were considered statistically significant if *P* < 0.05. For newly identified patients, genetic testing was performed by the Genetic Diagnostic Laboratory at Children's Hospital, Zhejiang University School of Medicine (Hangzhou, Zhejiang, China). Targeted next-generation sequencing (NGS) was performed as previously described [30]. All potentially pathogenic variants identified through NGS were validated by Sanger sequencing. One hundred healthy newborns who were negative for BKTD during screening were selected to assess variant frequencies in normal controls. The pathogenicity of novel variants was predicted using several in silico tools, including SIFT, PolyPhen-2, PROVEAN, and MutationTaster. To build three-dimensional (3D) models of ACAT1, homology modeling was employed using the Swiss Model Workspace (THIL_HUMAN P24752 Acetyl-CoA acetyltransferase, mitochondrial; Model 01), and PDB files were then submitted to Swiss-Pdb Viewer 4.10 for 3D-structure analysis. The color in this figure is selected by the Secondary Structure Succession of Swiss-Pdb Viewer 4.10.

## Supplementary Information

**Additional file 1. Table S1:** Biochemical, clinical, and molecular features of 29 Chinese patients with BKTD.

**Additional file 2. Table S2:** Biochemical features of BKTD patients identified by NBS and SMS.

**Additional file 3. Table S3:** In silico prediction and analysis of novel variants detected in ACAT1.

## Data Availability

The datasets used and/or analyzed during the current study can be obtained from the corresponding author upon reasonable request.

## Abbreviations

BKTD: Beta-ketothiolase deficiency
T2: Mitochondrial acetoacetyl-CoA thiolase
2M3HB: 2-Methyl-3-hydroxybutyrate
TIG: Tiglylglycine
2MAA: 2-Methylacetoacetate
NBS: Newborn screening
C5OH: 3-Hydroxyisovalerylcarnitine
C5:1: Tiglylcarnitine
SMS: Selective metabolic screening
NGS: Next-generation sequencing
3D: Three-dimensional
C4OH: 3-Hydroxybutyrylcarnitine
FN: False negatives
HSD10MD: HSD10 mitochondrial disease
