# Supplementary material for: C4OH is a potential newborn screening marker—a multicenter retrospective study of patients with beta-ketothiolase deficiency in China
Source: Orphanet J Rare Dis. 2021 May 17;16:224. doi: 10.1186/s13023-021-01859-5 (PMC8130433; doi:10.1186/s13023-021-01859-5)
Supplement: Supplementary file 1 — Additional file 1. Table S1: Biochemical, clinical, and molecular features of 29 Chinese patients with BKTD. [file 13023_2021_1859_MOESM1_ESM.docx]

**Table S1.** Biochemical, clinical, and molecular features of 29 Chinese BKTD patients

| Patient | Sex | Type | Age^e^ | Acylcarnitine analysis^f^ | | | Urinary organic acids | | Genotype | | Onset | Clinical manifestations | Precipitating factor | Outcome | References |
| --- | --- | --- | --- | --- | --- | --- | --- | --- | --- | --- | --- | --- | --- | --- | --- |
|  |  |  |  | C4OH | C5OH | C5:1 | 2M3HB | TIG | allele 1 | allele 2 |  |  |  |  |  |
| 1 | M | NBS | 9 y, 6 m | 0.92 | 1.86 | 0.31 | 76 | 38 | c.642T>G (p.Y214*) | c.997G>C (p.A333P) | None | None | None | Good | This study |
| 2^a^ | M | NBS | 6 y, 7 m | 0.87 | 0.78 | 0.21 | 90.33 | 52 | c.419T>G (p.L140R) | c.622C>T (p.R208*) | None | None | None | Good | This study |
| 3^a^ | M | NBS | 2 y, 2 m | 2.5 | 0.99 | 0.28 | 103 | 43 | c.419T>G (p.L140R) | c.622C>T (p.R208*) | None | None | None | Good | This study |
| 4 | F | NBS | 2 y, 1 m | 2.19 | 0.45 | 0.03 | 28.2 | 24 | c.163T>A (p.P55I) | **c.1119dup (p.V374Sfs*86)** | 11 m | Fever, severe metabolic acidosis | Vaccination | Developmental delay | This study |
| 5 | F | SMS | N/A | 2.48 | 1.42 | 1.22 | 43.71 | 53.85 | c.121-3C>G | c.121-3C>G | 8 m | Fever, tachypnea, seizures, severe metabolic acidosis | Vaccination | Died | This study |
| 6 | F | NBS | 6 y, 5 m | 0.46 | 0.6 | 0.28 | 17.65 | 4.84 | c.419T>G (p.L140R) | c.997G>C (p.A333P) | None | None | None | Good | This study |
| 7 | M | NBS | 5 y, 1 m | 0.8 | 1.32 | 0.48 | 9.15 | 13.29 | c.1124A>G (p.N375S) | c.622C>T (p.R208*) | 3 d | Tachypnea, metabolic acidosis | Infection | Good | This study |
| 8^b^ | M | NBS | 1 y, 6 m | 1.02 | 0.87 | 0.27 | 360.31 | 44.45 | c.622C>T (p.R208*) | **c.631C>A (p.Q211K)** | None | None | None | Good | This study |
| 9^b^ | F | SMS | N/A | N/A | N/A | N/A | N/A | N/A | c.622C>T (p.R208*) | **c.631C>A (p.Q211K)** | 16 m | Hypotonia | N/A | Good | This study |
| 10 | F | NBS | 1 y, 8 m | 0.26 | 1.8 | 0.46 | 647.72 | 40.11 | c.622C>T (p.R208*) | **c.1154A>T (p.H385L)** | None | None | None | Good | This study |
| 11 | M | SMS | 3 y, 8 m | 1.15 | 0.44 | 0.02 | 130 | N/A | **c.401T>C (p.M134T)** | **c.481T>C (p.Y161H)** | 12 m | Tachypnea, severe metabolic acidosis | Infection | Died | This study |
| 12 | M | SMS | 3 y, 8 m | N/A | N/A | N/A | 21.1 | 150.4 | c.756_758del (p.E252del) | c.1006-1G>C | 9 m | Tachypnea, severe metabolic acidosis | Diarrhea | Died | This study |
| 13 | N/A | SMS | N/A | N/A | 1.3 | 0.2 | 103.87 | 127.01 | c.1124A>G (p.N375S) | c.1124A>G (p.N375S) | N/A | Neurological impairment | N/A | N/A | Xu et al. (2019) |
| 14 | N/A | SMS | N/A | N/A | 1.43 | 0.43 | 11.96 | 32.22 | c.229del (p.E77Kfs*10) | c.1124A>G (p.N375S) | N/A | Neurological impairment | N/A | N/A | Xu et al. (2019) |
| 15 | N/A | SMS | N/A | N/A | 1.43 | 0.43 | 182.2 | 116.35 | c.373G>T (p.V125F) | c.373G>T (p.V125F) | N/A | Neurological impairment | N/A | N/A | Xu et al. (2019) |
| 16 | N/A | NBS | N/A | N/A | 2.72 | 0.62 | 84.89 | 33.71 | c.419T>G (p.L140R) | c.997G>C (p.A333P) | None | None | None | Good | Xu et al. (2019) |
| 17^c^ | M | NBS | 5 y, 2 m | 1.49 | 0.78 | 0.14 | 5.36 | 0.6 | c.72+1G>A | exon 6-12del | None | None | None | Good | Xu et al. (2019) |
| 18^c^ | F | NBS | 5 y, 2 m | 1.5 | 0.85 | 0.16 | 9.4 | 0.58 | c.72+1G>A | exon 6-12del | None | None | None | Good | Xu et al. (2019) |
| 19 | N/A | NBS | N/A | N/A | 3.4 | 0.89 | 27.18 | 54.45 | c.622C>T (p.R208*) | c.1124A>G (p.N375S) | None | None | None | Good | Xu et al. (2019) |
| 20^d^ | M | SMS | N/A | 0.79 | 2.02 | 0.4 | 15.44 | 27.72 | c.622C>T (p.R208*) | c.653C>T (p.S218F) | 13 m | Fever, vomiting, severe ketoacidosis | N/A | N/A | Wen et al. (2016) |
| 21^d^ | M | SMS | N/A | 0.79 | 2.02 | 0.4 | 15.44 | 27.72 | c.622C>T (p.R208*) | c.653C>T (p.S218F) | 13 m | Fever, vomiting, severe ketoacidosis | N/A | N/A | Wen et al. (2016) |
| 22^d^ | F | SMS | N/A | N/A | N/A | N/A | N/A | N/A | c.622C>T (p.R208*) | c.653C>T(p.S218F) | 5 m | Severe ketoacidosis | N/A | Good | Wen et al. (2016) |
| 23 | M | NBS | N/A | 3.58 | 1.31 | 0.31 | 4.14 | 9.8 | c.721dup (p.T241Nfs*14) | c.928G>C (p.A310P) | 8 d | Tachypnea,metabolic acidosis | Pulmonary infection | Good | Yang et al. (2019) |
| 24 | M | NBS | N/A | 1.22 | 0.71 | 0.2 | 14.08 | 5.74 | c.238+1G>A | c.1163G>T (p.G388V) | None | None | None | Good | Yang et al. (2019) |
| 25 | M | SMS | N/A | N/A | ↑↑↑ | ↑↑↑ | ↑↑↑ | ↑↑ | c.829A>C (p.T277P) | c.997G>C (p.A333P) | 8 m | Tachypnea, kussmaul respiration, seizures, and severe metabolic acidosis | N/A | Good | Su et al. (2017) |
| 26 | F | SMS | N/A | N/A | ↑↑ | ↑↑ | ↑↑↑ | ↑↑↑ | c.890C>A (p.T297K) | c.121-3C>G | 8 m | Tachypnea, vomiting, exhaustion, and severe metabolic acidosis | N/A | Good | Su et al. (2017) |
| 27 | F | SMS | N/A | N/A | ↑↑↑ | ↑↑↑ | ↑↑ | ↑↑↑ | c.83_84del (p.Y28Cfs*38) | c.1006-1G>C | 13 m | Coma, seizures, and refractory metabolic acidosis | Diarrhea | Good | Su et al. (2017) |
| 28 | M | SMS | N/A | N/A | ↑↑↑ | ↑↑↑ | ↑↑↑ | ↑↑ | c.1006-1G>C | c.1006-1G>C | 9 m | Severe ketoacidosis | Infection | Good | Su et al. (2017) |
| 29 | M | SMS | N/A | N/A | N/A | N/A | ↑↑↑ | ↑↑↑ | c.1006-1G>C | c.354_355delinsG (p.C119Vfs*4) | 12 m | Fever, vomiting, impaired consciousness, severe ketoacidosis | Infection | N/A | Law et al. (2015) |

a,b,c,d: siblings; e: age as of December 2020; d: day, m: month, y: year; M: male, F: female; NBS: newborn screening, SMS: selective metabolic screening; f: data from SMS represent the acylcarnitine concentrations identified at the time of acute decompensation; N/A: not available; C4OH: 3-hydroxybutyrylcarnitine, reference value: 0.02-0.3μmol/L, C5OH: 3-hydroxyisovalerylcarnitine, reference value: 0.06-0.5 μmol/L, C5:1: tiglylcarnitine, reference value: 0-0.05 μmol/L; 2M3HB: 2-methyl-3-hydroxybutyrate, reference value: 0-0.3, TIG: tiglylglycine: 0-0.1; ↑↑↑: massive elevation, ↑↑: medium elevation.

The previously unreported novel variants of this study are in boldface type.
