# Supplementary material for: C4OH is a potential newborn screening marker—a multicenter retrospective study of patients with beta-ketothiolase deficiency in China
Source: Orphanet J Rare Dis. 2021 May 17;16:224. doi: 10.1186/s13023-021-01859-5 (PMC8130433; doi:10.1186/s13023-021-01859-5)
Supplement: Supplementary file 2 — Additional file 2. Table S2: Biochemical features of BKTD patients identified by NBS and SMS. [file 13023_2021_1859_MOESM2_ESM.docx]

**Table S2.** Biochemical features of BKTD patients identified by NBS and SMS

| NBS | | | | SMS | | | |
| --- | --- | --- | --- | --- | --- | --- | --- |
| Patient No. | C4OH | C5OH | C5:1 | Patient No. | C4OH | C5OH | C5:1 |
| 1 | 0.92 | 1.86 | 0.31 | 5 | 2.48 | 1.42 | 1.22 |
| 2 | 0.87 | 0.78 | 0.21 | 9 | N/A | N/A | N/A |
| 3 | 2.5 | 0.99 | 0.28 | 11 | 1.15 | 0.44 | 0.02 |
| 4 | 2.19 | 0.45 | 0.03 | 12 | N/A | N/A | N/A |
| 6 | 0.46 | 0.6 | 0.28 | 13 | N/A | 1.3 | 0.2 |
| 7 | 0.8 | 1.32 | 0.48 | 14 | N/A | 1.43 | 0.43 |
| 8 | 1.02 | 0.87 | 0.27 | 15 | N/A | 1.43 | 0.43 |
| 10 | 0.26 | 1.8 | 0.46 | 20 | 0.79 | 2.02 | 0.4 |
| 16 | N/A | 2.72 | 0.62 | 21 | 0.79 | 2.02 | 0.4 |
| 17 | 1.49 | 0.78 | 0.14 | 22 | N/A | N/A | N/A |
| 18 | 1.5 | 0.85 | 0.16 | 25 | N/A | ↑↑↑ | ↑↑↑ |
| 19 | N/A | 3.4 | 0.89 | 26 | N/A | ↑↑ | ↑↑ |
| 23 | 3.58 | 1.31 | 0.31 | 27 | N/A | ↑↑↑ | ↑↑↑ |
| 24 | 1.22 | 0.71 | 0.2 | 28 | N/A | ↑↑↑ | ↑↑↑ |
|  |  |  |  | 29 | N/A | N/A | N/A |
| Median | 1.40 | 2.72 | 2.39 |  | 2.09 | 3.53 | 3.25 |
| Median (NBS plus SMS) | 1.38 | 1.36 | 0.37 |  |  |  |  |

NBS: newborn screening, SMS: selective metabolic screening, N/A: not available.

C4OH: 3-hydroxybutyrylcarnitine, reference value: 0.02-0.3 μmol/L, C5OH: 3-hydroxyisovalerylcarnitine, reference value: 0.06-0.5 μmol/L, C5:1: tiglylcarnitine, reference value: 0-0.05 μmol/L.

↑↑↑: massive elevation, ↑↑: medium elevation.
