# Supplementary material for: C4OH is a potential newborn screening marker—a multicenter retrospective study of patients with beta-ketothiolase deficiency in China
Source: Orphanet J Rare Dis. 2021 May 17;16:224. doi: 10.1186/s13023-021-01859-5 (PMC8130433; doi:10.1186/s13023-021-01859-5)
Supplement: Supplementary file 3 — Additional file 3. Table S3: In silico prediction and analysis of novel variants detected in ACAT1. [file 13023_2021_1859_MOESM3_ESM.docx]

**Table S3:** *In silico* prediction and analysis of the novel variants detected in *ACAT1*

| No. | Variants | dbSNP^a^ | Freq in ExAC^b^ | Freq in GnomAD^c^ | Freq in 1000 Genome^d^ | SIFT (score)^e^ | PolyPhen-2 (score)^f^ | PROVEAN (score)^g^ | Mutation Taster (score)^h^ |
| --- | --- | --- | --- | --- | --- | --- | --- | --- | --- |
| 1 | c.401T>C (p.M134T) | rs779097442 | 8.00E-06 | 4.06E-06 | ND | 0.034 | 0.989 | -5.49 | 1 |
| 2 | c.481T>C (p.Y161H) | rs768121096 | ND | 4.06E-06 | ND | 0 | 0.998 | -4.44 | 1 |
|  | c.631C>A (p.Q211K) | ND | ND | ND | ND | 0 | 0.998 | -4.00 | 0.999 |
| 4 | c.1119dup (p.V374Sfs*86) | ND | ND | ND | ND | N/A | N/A | N/A | 1 |
| 5 | c.1154A>T (p.H385L) | ND | ND | ND | ND | 0 | 1 | -11.00 | 0.999 |

ND: no data.

N/A: not available.

^a^dbSNP: <https://www.ncbi.nlm.nih.gov/projects/SNP/>, ^b/c^ExAC/GnomAD: <http://gnomad.broadinstitute.org/>, ^d^1000 Genome Project: <http://www.1000genomes.org/>.

^e^SIFT: <http://sift.jcvi.org/>, ^f^PolyPhen-2: <http://genetics.bwh.harvard.edu/pph2/>, ^g^PROVEAN: <http://provean.jcvi.org/index.php>, ^h^MutationTaster: <http://www.mutationtaster.org/>.
